# *Aurkb* deficiency disrupts microglial development, homeostasis and hinders remyelination following cuprizone-induced demyelination

**DOI:** 10.1016/j.isci.2026.114718

**Published:** 2026-01-20

**Authors:** Weixing Yan, Dong Xiang, Li Du, Di Zhu, Qi Jia, Yuting Liu, Siyu Wang, Li Liu, Haihao Guan, Yelin Zhao, Guan Jiang, Sijia Gao, Hui Wang

**Affiliations:** 1 Jiangsu Key Laboratory of Immunity and Metabolism, Department of Pathogen Biology and Immunology, School of Basic Medical Science, Xuzhou Medical University, Xuzhou, Jiangsu 221004, China; 2 National Experimental Demonstration Center for Basic Medicine Education, Xuzhou Medical University, Xuzhou, Jiangsu 221004, China; 3 Department of Dermatology, The Affiliated Hospital of Xuzhou Medical University, Xuzhou, Jiangsu 221002, China; 4 Department of Neurology, The Affiliated Hospital of Xuzhou Medical University, Xuzhou, Jiangsu 221006, China; 5 Department of Clinical Science, Intervention and Technology, Karolinska Institutet, 17165 Stockholm, Sweden; 6 Department of Clinical Laboratory, Xuzhou Maternity and Child Health Hospital, Xuzhou, Jiangsu 221004, China

**Keywords:** Neuroscience, Cell biology

## Abstract

Microglia are crucial for phagocytic clearance of myelin debris, which hinders remyelination and leads to neurological decline during aging and in multiple sclerosis (MS). However, the molecular mechanism enabling microglia to expand and function effectively in remyelination remains elusive. Here, we identified that mitotic kinase Aurkb was upregulated in microglia during early development and in MS. Neonatal deletion of *Aurkb* disrupted cell density, morphology, and proliferation, which is attributed to stalled mitosis. Inducible *Aurkb* ablation in adulthood led to microglial dystrophy and disrupted homeostasis. *Aurkb* deficiency compromised microglial activation in response to LPS-induced inflammation. Critically, *Aurkb*-deficient mice exhibited accumulated myelin debris and impaired oligodendrocyte regeneration and remyelination in the CPZ-induced demyelination model. Additionally, *Aurkb* deletion inhibited microglial clearance of myelin debris, independent of reduced microglia numbers. This defect was associated with diminished autophagy. Together, these findings establish Aurkb as a key regulator of microglial development, homeostasis, and responses to remyelination.

## Highlights

• Aurkb increases in a subset of microglia during early development and demyelination
• Neonatal *Aurkb* ablation impairs microglial mitosis, density, and morphology
• *Aurkb* loss in adulthood disrupts microglial homeostasis, activation, and autophagy
• *Aurkb* deficiency limits microglial phagocytic function and hinders remyelination

## Introduction

Microglia, the resident macrophages of the central nervous system (CNS), are essential for brain development, homeostasis and response to CNS insults. Microglia proliferate and self-renew locally, enabling the establishment of the adult population during early development and rapid expansion in response to CNS damage. In the context of normal aging and multiple sclerosis (MS), a chronic immune-mediated CNS demyelinating disease,^1^ toxic myelin debris hinders oligodendrocyte regeneration, ultimately leading to impaired remyelination and neurological decline.^2^ In response, microglia proliferate and accumulate within demyelinating lesions, where they phagocytose myelin debris, creating a permissive environment for oligodendrocyte regeneration and remyelination.^3^^,^^4^ Despite the identification of multiple mediators regulating microglial responses during remyelination,^5^^,^^6^^,^^7^^,^^8^^,^^9^ the mechanisms underlying microglial expansion and functional responses in this context remain poorly understood.

Microglia originate from yolk sac-derived myeloid progenitors, with their development beginning before embryonic day 8 (E8). Between E9.5 and E14.5, these progenitors migrate into the developing brain.^10^^,^^11^^,^^12^ Postnatally, microglia undergo a phase of rapid expansion within the first two weeks, followed by a gradual decline until adulthood.^13^^,^^14^ Adult microglia are long-lived and self-maintained with a density at approximately 50% of the peak levels reached during the second postnatal week.^15^^,^^16^ Under homeostasis, microglia exhibit a ramified morphology and actively survey the CNS microenvironment, but rapidly shift to an amoeboid and phagocytic phenotype in response to neurodegenerative insults or injury.^2^^,^^16^

It is reported that microglia during early development and responses to demyelination exhibit an activated cell cycle pathway.^13^^,^^14^^,^^17^ Aurora kinase B (Aurkb), a key component of the chromosomal passenger complex, increases in G_2_/M and controls mitosis by regulating chromosome segregation and cytokinesis.^18^^,^^19^^,^^20^^,^^21^ Despite this, previous studies show that Aurkb is also expressed during G_0_/G_1_ in quiescent cells and regulates lymphocyte survival and maintenance.^22^^,^^23^ Additionally, Aurkb has been implicated in regulating mTOR signaling,^23^ a key pathway that modulates autophagy, which in turn is essential for microglial phagocytic function in neurodegenerative diseases.^24^^,^^25^ In a rat model of nerve injury, the inhibition of Aurkb activity attenuates spinal microgliosis.^26^ Nevertheless, the function of Aurkb in microglial development, homeostasis, and responses to remyelination remains elusive.

In this study, we examined Aurkb expression in microglia during development and following cuprizone (CPZ)-induced demyelination. To investigate its role, we employed microglia-specific *Aurkb* knockout mice in combination with a CPZ-induced demyelination model. Our results demonstrate that Aurkb is critical for microglial development, homeostasis, and efficient clearance of myelin debris, revealing its previously unrecognized role in CNS repair.

## Results

### Induced aurora kinase B expression in mouse microglia during early development and aurora kinase B expression in human microglia in multiple sclerosis

**Figure 1:**
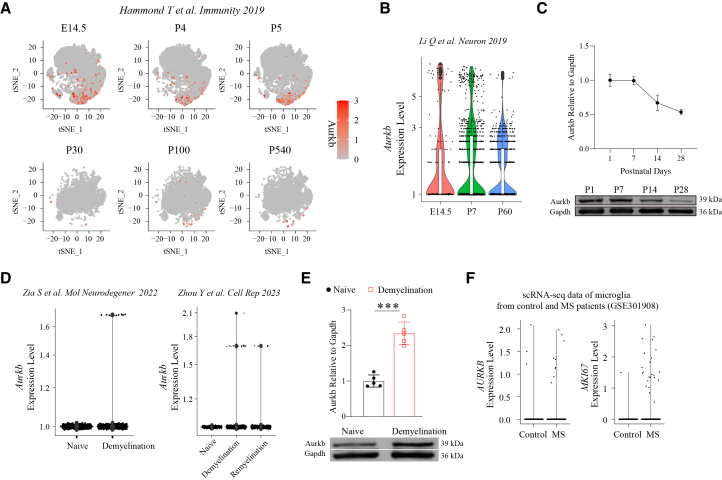
Induced Aurkb expression in mouse microglia during early development and *AURKB* expression in human microglia in multiple sclerosis (A) t-SNE plot of scRNA-seq data (GSE121654) showing *Aurkb* expression in mouse microglia across various developmental stages. Embryonic day 14.5, E14.5; Postnatal, P. (B) Violin plot from scRNA-seq data (GSE123025) depicting *Aurkb* expression in microglia from E14.5, P7, and P60. (C) Representative Western blot images (upper panel) and quantification (lower panel) of Aurkb expression in microglia at P1, P7, P14, and P28 (*n* = 3 mice per time point). (D) Violin plot from scRNA-seq data (GSE207570, GSE204755) depicting *Aurkb* expression in microglia from naive and CPZ-treated mice across demyelination and remyelination stages. (E) Representative Western blot images (upper panel) and quantification plot (lower panel) of Aurkb expression in microglia from naive and CPZ-treated mice during the demyelination stage (*n* = 5 mice per group). f) Violin plot shows the expression of *AURKB* and *MKI67* in microglia from control and multiple sclerosis (MS) cohorts (public scRNA-seq dataset GSE301908). Data are presented as the mean ± SD; Two-tailed unpaired t-tests in (E). ∗∗∗*p* < 0.001 compared with the naive group. See also Figure S1. See Data S1 for uncropped blots.

In an analysis of two independent scRNA-seq datasets of microglia from different developmental stages in mice, we identified that *Aurkb* is highly expressed in a subset of fetal and neonatal microglia (Figures 1A and 1B).^27^^,^^28^ Of note, increased *Aurkb* coincides with the proliferation marker *Mki67* (Figure S1A). Consistently, Western blot analysis revealed that Aurkb expression in microglia decreased progressively from postnatal day 7–28 (Figure 1C). To determine whether Aurkb expression was upregulated in microglia under demyelinating conditions, we examined public scRNA-seq data from the CPZ-induced demyelination model. A minor subpopulation of microglia exhibited high Aurkb expression, which was further confirmed in an independent scRNA-seq dataset covering both demyelination and remyelination phases^29^ (Figure 1D). In line with this, Western blot analysis confirmed increased Aurkb expression in microglia following CPZ-induced demyelination (Figure 1E). Furthermore, analysis of public scRNA-seq data of microglia from MS cohorts and controls revealed increased expression of proliferation marker *MKI67* and *AURKB* in MS samples (Figures 1F and S1B). These findings indicate that Aurkb may be involved in regulating microglial development and responses to demyelination and remyelination in MS.

### Aurora kinase B deficiency reduces microglial density and induces dystrophy in adult mice

**Figure 2:**
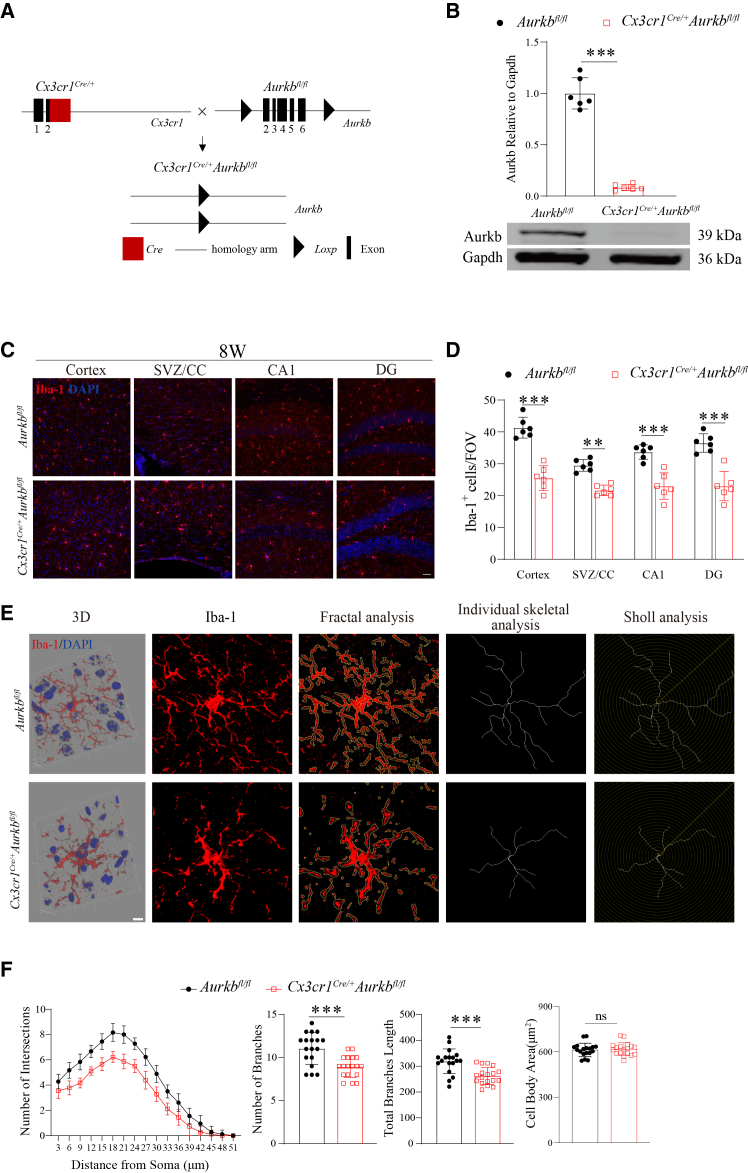
*Aurkb* deficiency reduces microglial density and induces dystrophy in adult mice (A) Schematic of microglial *Aurkb* conditional knockout strategy. (B and C) Representative Western blot images (upper panel) and quantification (lower panel) of Aurkb expression in microglia (*n* = 6 mice per genotype); (C) representative immunofluorescence and d) quantification of microglial density (Iba-1^+^) across CNS regions from 8-week-old (8W) *Aurkb*^*fl/fl*^ and *Cx3cr1*^*Cre/+*^*Aurkb*^*fl/fl*^ mice (*n* = 6 mice per genotype, Scale bars: 50 μm). SVZ, subventricular zone; CC, corpus callosum; CA, Cornu Ammonis; DG, dentate gyrus. (E–F) Representative images and quantification of microglial processes and branch intersections by combined Sholl, skeletal, and fractal analysis (*n* = 6 mice per genotype, Scale bars:10 μm). Three microglia per mouse were quantified. Data are presented as the mean ± SD; Two-tailed unpaired t-tests in (B); two-way ANOVA with Bonferroni multiple comparisons test in (D); linear mixed-effects models for continuous data and negative binomial generalized linear mixed-effects models for count data, with repeated measures from the same mouse accounted for as a random effect, followed by Tukey-adjusted pairwise tests in (F); ∗∗*p* < 0.01, ∗∗∗*p* < 0.001; ns, not significant compared with the *Aurkb*^*fl/fl*^ group. See also Figure S2. See Data S1 for uncropped blots.

To elucidate the role of Aurkb in microglial development, we generated *Aurkb* conditional knockout mice using CRISPR-Cas9-mediated genome editing. We engineered a conditional knockout allele by flanking exons 2–6 of *Aurkb* with *LoxP* sites (Figure 2A). These *Aurkb*-floxed mice were then crossed with *Cx3cr1*^*Cre/+*^ mice, which express Cre recombinase specifically in microglia, to generate microglia-specific knockout mice, *Cx3cr1*^*Cre/+*^*Aurkb*^*fl/fl*^. In this model, Cre-mediated recombination results in the excision of exons 2–6, leading to a complete loss of functional Aurkb protein expression, as confirmed by Western blot analysis (Figure 2B).

Immunofluorescence analysis revealed a significant reduction in microglial density across multiple CNS regions, including cortex, subventricular zone (SVZ), corpus callosum (CC), Cornu Ammonis (CA1), and dentate gyrus (DG) in adult *Cx3cr1*^*Cre/+*^*Aurkb*^*fl/fl*^ mice (Figures 2C, 2D, and S2). Furthermore, *Aurkb*-deficient microglia displayed pronounced morphological abnormalities, exhibiting a dystrophic phenotype characterized by markedly reduced branching complexity and significantly shorter processes (Figures 2E and 2F). These findings demonstrate that Aurkb is essential for maintaining adult microglial population density and ramified morphology.

### Neonatal *Aurkb* deficiency disrupts microglial development by impairing infant microglial proliferation and survival in a mitosis-kinase-dependent manner

**Figure 3:**
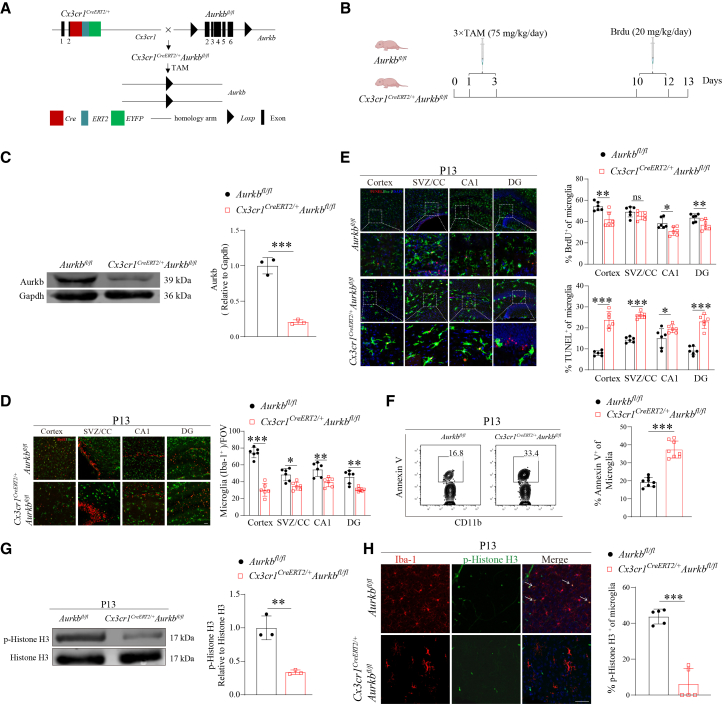
Neonatal *Aurkb* deletion disrupts microglial development (A) Schematic of tamoxifen (TAM)-inducible microglial *Aurkb* knockout strategy. (B) Experimental timeline: TAM injections at P1-P3, BrdU pulses at P10 (every 24 h for 3 days), and tissue collection at P13. (C) Western blot (left panel) and quantification (right panel) of Aurkb in microglia (*n* = 3 mice per genotype). (D) Representative immunofluorescence images (left panel) and quantification (right panel) of microglial density (Iba-1^+^) across CNS regions at P13 (*n* = 6 mice per genotype, Scale bars: 50 μm). (E) Representative immunofluorescence images (left panel) and corresponding quantification (right panel) of BrdU^+^ (proliferation) and TUNEL^+^ (apoptosis) microglia (Iba-1^+^) at P13 (*n* = 6 mice per genotype). Scale bars: 50 μm). (F) Representative flow cytometric images (left panel) and quantification (right panel) of apoptotic microglia (Annexin V^+^) at P13 (*n* = 8 mice per genotype). (G and H) Western blot (*n* = 3 mice per genotype) and H) immunofluorescence analyses of phospho-histone H3 (pH3) in microglia (Iba-1^+^) at P13 (*n* = 5 mice per genotype, Scale bars: 50 μm). Data are presented as the mean ± SD. Two-tailed unpaired t-tests in (C, F, G, and H); two-way ANOVA with Bonferroni multiple comparisons test in (D and E); ∗*p* < 0.05, ∗∗*p* < 0.01, ∗∗∗*p* < 0.001; ns, not significant compared with the *Aurkb*^*fl/fl*^ group. See also Figure S3. See Data S1 for uncropped blots.

In adult brains, microglia undergo slow self-renewal, whereas neonatal mice exhibit a burst of microglial proliferation within the first 1–2 postnatal weeks.^30^ Given the high expression of Aurkb in a subset of embryonic and neonatal microglia, we examined its role in microglial development during this critical window. To this end, we generated tamoxifen-inducible, microglial-specific knockout mice *Cx3cr1*^*CreERT2/+*^*Aurkb*^*fl/fl*^, which allow spatiotemporal control of *Aurkb* deletion (Figure 3A). Consecutive tamoxifen administration on postnatal days 1–3 efficiently ablated *Aurkb* in microglia (Figures 3B and 3C).

To assess the impact of *Aurkb* loss in neonatal microglia, we analyzed microglial abundance, proliferation, and apoptosis in 13-day-old *Aurkb*^*fl/fl*^ and *Cx3cr1*^*CreERT2/+*^ *Aurkb*^*fl/fl*^ mice. Conditional deletion of *Aurkb* in microglia significantly reduced microglial density across neonatal brain regions (Figures 3D and S3). The BrdU incorporation assay showed decreased microglial proliferation in the cerebral cortex and hippocampus (Figures 3E–3D and S3). Since the upregulation of Aurkb is essential for mitosis, and defective mitosis can trigger apoptosis,^31^^,^^32^ we next assessed microglial death using TUNEL staining. This analysis revealed increased microglial death in the cortex, SVZ/CC, and hippocampus of *Cx3cr1*^*CreERT2/+*^*Aurkb*^*fl/fl*^ mice (Figures 3E and S3). These findings were further supported by flow cytometric analysis, which confirmed elevated apoptotic microglia in *Aurkb*-deficient mice (Figure 3F).

Aurkb facilitates cell proliferation by phosphorylating histone H3,^33^^,^^34^ a modification critical for mitotic progression. To determine whether this mechanism is active in neonatal microglia, we assessed phospho-histone H3 levels in purified neonatal microglia. Western blot analysis revealed a marked reduction in phospho-histone H3 in *Aurkb*-deficient microglia (Figure 3G). This finding was corroborated by immunofluorescence, which showed strong co-localization of microglial marker Iba-1 and p-histone H3 in wild-type microglia, but minimal signal in *Aurkb*-deficient microglia (Figure 3H).

Collectively, these results demonstrate that Aurkb is essential for maintaining neonatal microglial development by promoting proliferation and preventing apoptosis in a mitosis-kinase-dependent manner.

### Tamoxifen-induced aurora kinase B deletion in adult mice impairs microglial homeostasis

Aurkb is also expressed in quiescent cells and regulates the survival and maintenance of quiescent lymphocytes.^22^^,^^23^ To assess whether Aurkb controls microglial homeostasis, we induced *Aurkb* deletion in adult *Aurkb*^*fl/fl*^ mice and *Cx3cr1*^*CreERT2/+*^*Aurkb*^*fl/fl*^ mice by administering tamoxifen for five consecutive days. Microglial morphology and dynamics were analyzed at 1-month and 3-month post-tamoxifen.

**Figure 4:**
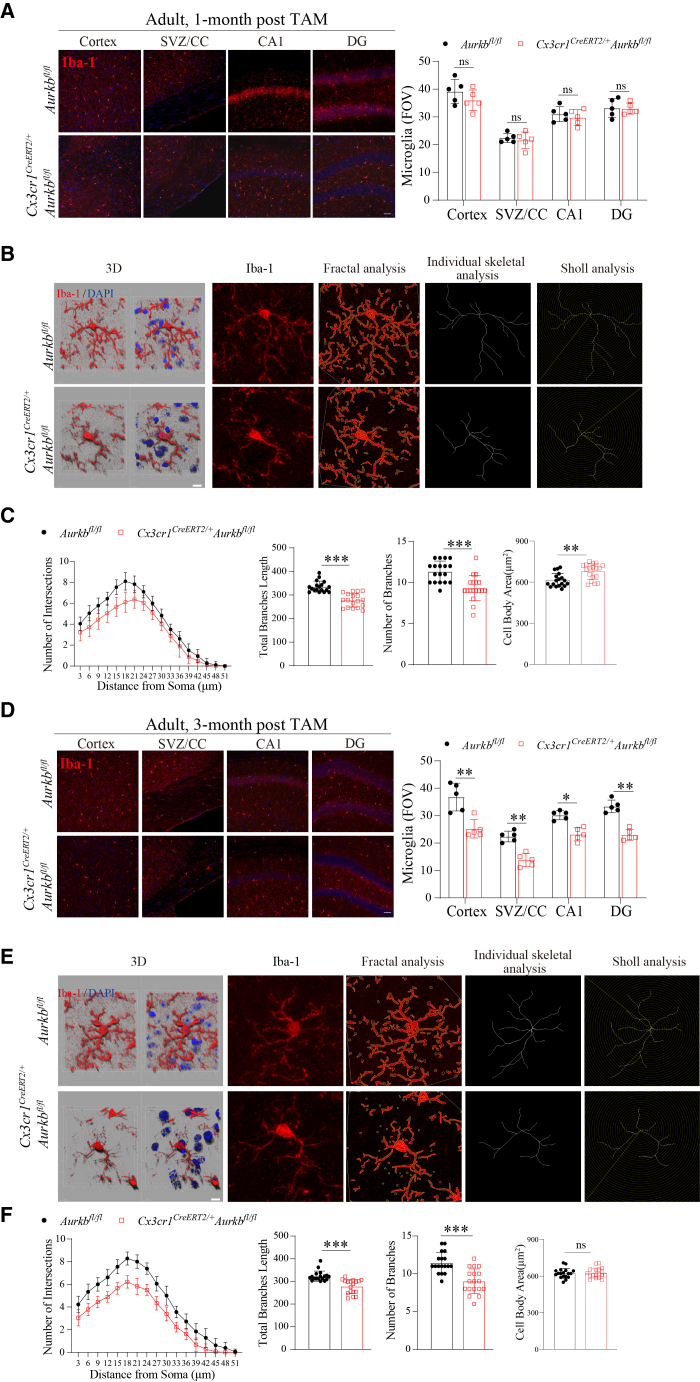
Inducible *Aurkb* ablation in adult mice impairs microglial homeostasis (A–F) Adult *Aurkb*^*fl/fl*^ and *Cx3cr1*^*CreERT2/+*^*Aurkb*^*fl/fl*^ littermates (8-week-old) were *i.p.* injected with TAM for 5 consecutive days, followed by tissue collection at 1-month and 3-month post TAM induction. Representative immunofluorescence and quantification of microglial density (Iba-1^+^) across CNS regions at A) 1-month and D) 3-month post-tamoxifen induction (*n* = 5 mice per genotype, Scale bars: 50 μm). The morphology analysis of microglial processes and branch intersections by combined Sholl, skeletal, and fractal analysis. (B–C) 1-month and e-f) 3-month post-TAM induction (*n* = 5 mice per genotype, Scale bars: 10 μm). Data are presented as the mean ± SD. two-way ANOVA with Bonferroni multiple comparisons test in (A and D); linear mixed-effects models for continuous data and negative binomial generalized linear mixed-effects models for count data, with repeated measures from the same mouse accounted for as a random effect, followed by Tukey-adjusted pairwise tests in (C–F); ∗*p* < 0.05, ∗∗*p* < 0.01, and ∗∗∗*p* < 0.001; ns, not significant compared with the *Aurkb*^*fl/fl*^ group. See also Figure S4.

At 1-month post-induction, microglial density remained largely unchanged in *Aurkb*-deficient mice (Figures 4A and S4A), consistent with the slow turnover and homeostatic stability of adult microglia. Despite this, microglial morphology analysis by combined skeletal, fractal, and Sholl analysis revealed that *Aurkb* deletion in adult microglia led to microglial dystrophy characterized by reduced branching complexity and shortened processes (Figures 4B and 4C). In contrast, by 3-month post-induction, a significant reduction in microglial density was observed in *Cx3cr1*^*CreERT2/+*^*Aurkb*^*fl/fl*^ mice (Figures 4D and S4B), indicating progressive microglial loss. Similarly, *Aurkb*-deficient microglia following 3-month tamoxifen induction exhibit dystrophic morphology (Figures 4E and 4F).

These findings suggest that *Aurkb* is required for the long-term self-maintenance of adult microglia.

### Aurora kinase B deficiency leads to transient CD68 upregulation in homeostatic microglia but attenuates its induction during LPS-induced inflammation

**Figure 5:**
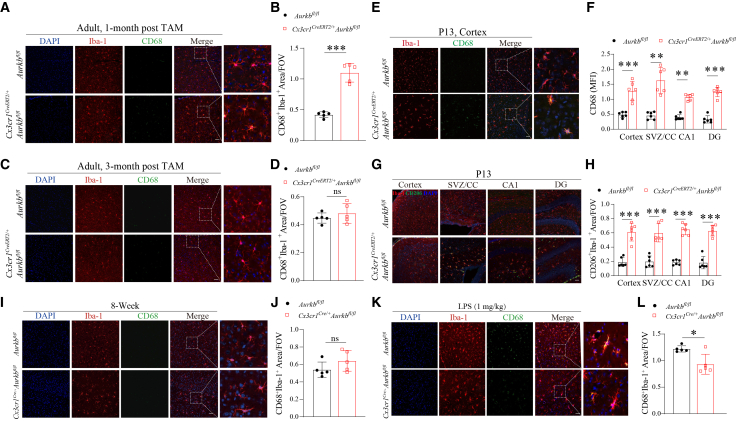
*Aurkb* loss transiently elevates CD68 in homeostatic microglia but compromises its upregulation in an LPS-induced inflammation model (A–D) Adult *Aurkb*^*fl/fl*^ and *Cx3cr1*^*CreERT2/+*^*Aurkb*^*fl/fl*^ littermates (8-week-old) were *i.p.* injected with TAM for 5 consecutive days, followed by tissue collection at 1-month and 3-month post TAM induction. Representative immunofluorescence and quantification of CD68 in microglia at (A and B) 1-month and (C and D) 3-month post-TAM induction (*n* = 5 mice per genotype per time point, Scale bars: 50 μm). (E–H) Neonatal *Aurkb*^*fl/fl*^ and *Cx3cr1*^*CreERT2/+*^*Aurkb*^*fl/fl*^ littermates were *i.p.* injected with TAM for 3 consecutive days at P1-P3, followed by tissue collection at P13. Representative immunofluorescence and quantification of (E and F) CD68 and (G and H) CD206 in microglia at P13 (*n* = 6 mice per genotype, Scale bars: 50 μm). (I and J) Representative immunofluorescence and quantification of CD68 in microglia from adult *Aurkb*^*fl/fl*^ and *Cx3cr1*^*Cre/+*^*Aurkb*^*fl/fl*^ littermates (*n* = 5 mice per genotype, Scale bars: 50 μm). The representative immunofluorescence image of the *Cx3cr1*^*Cre/+*^*Aurkb*^*fl/fl*^ group is shared in Figure 2C. (K and L) Adult *Aurkb*^*fl/fl*^ and *Cx3cr1*^*Cre/+*^*Aurkb*^*fl/fl*^ littermates were *i.p.* injected with LPS (1 mg/kg) and sacrificed at 48 h post LPS administration (*n* = 5 mice per genotype, Scale bars: 50 μm). Data are presented as the mean ± SD. Two-tailed unpaired t-tests in (B, D, J, and I); two-way ANOVA with Bonferroni multiple comparisons test in (F, H); ∗*p* < 0.05, ∗∗*p* < 0.01, ∗∗∗*p* < 0.001. ns, not significant compared with the *Aurkb*^*fl/fl*^ group. See also Figures S5 and S6.

Microglial activation is characterized by increased CD68 expression accompanied by morphological changes, including soma enlargement and process thickening.^35^^,^^36^ Since *Aurkb* deficiency led to enlarged somata, shortened and thickened processes (Figures 2E and 4B), we next examined whether *Aurkb* deficiency induced microglial activation. To achieve this, we assessed the expression of CD68, a canonical marker for activated microglia. Immunofluorescence analysis revealed significant upregulation of CD68 in microglia from *Cx3cr1*^*CreERT2/+*^*Aurkb*^*fl/fl*^ mice 1-month after tamoxifen induction (Figures 5A and 5B). However, this effect is transient, as CD68 expression reverted to baseline by three months post-induction (Figures 5C and 5D). To further validate this transient activation, we analyzed neonatal microglia following the tamoxifen-induced deletion of *Aurkb.* Both immunofluorescence and flow cytometric analyses confirmed robust CD68 upregulation in *Aurkb*-deficient infant microglia (Figures 5E, 5F, S5A, and S5B). In addition, CD206, typically expressed by perivascular macrophages and leptomeningeal macrophages but also upregulated in reactive microglia,^37^^,^^38^ was upregulated in *Aurkb*-deficient infant microglia across multiple CNS regions (Figures 5G and 5H). In contrast, CD68 expression remained unchanged in adult *Cx3cr1*^*Cre/+*^*Aurkb*^*fl/fl*^ mice (Figures 5I and 5J), indicating that *Aurkb* deficiency in microglia leads to a transient activation phenotype under homeostatic conditions.

Interestingly, this transient CD68 upregulation contrasts with a previous report that the pharmacological inhibition of Aurkb limits microglial activation in a neuropathic pain model.^26^^,^^39^ To further understand the role of Aurkb in microglia activation, we employed an LPS-induced inflammation model,^40^^,^^41^ in which microglia undergo rapid proliferation and activation associated with CD68 upregulation and morphological changes from a ramified to an amoeboid-like form. While wild-type microglia showed typical activation following LPS challenge, *Aurkb*-deficient microglia largely retained a resting morphology with compromised CD68 upregulation (Figures 5K and 5L). However, single-cell confocal imaging revealed post-LPS morphological abnormalities in *Aurkb*-deficient microglia, including enlarged somata and frequent signs of cytokinesis failure (Figure S6), suggesting mitotic dysregulation and impaired capacity to mount an effective inflammatory response. Altogether, *Aurkb* deficiency leads to transient microglia activation under homeostatic conditions but attenuates its activation during LPS-induced inflammation.

### Aurora kinase B deficiency in microglia impairs remyelination and oligodendrocyte regeneration in the cuprizone-induced demyelination model

**Figure 6:**
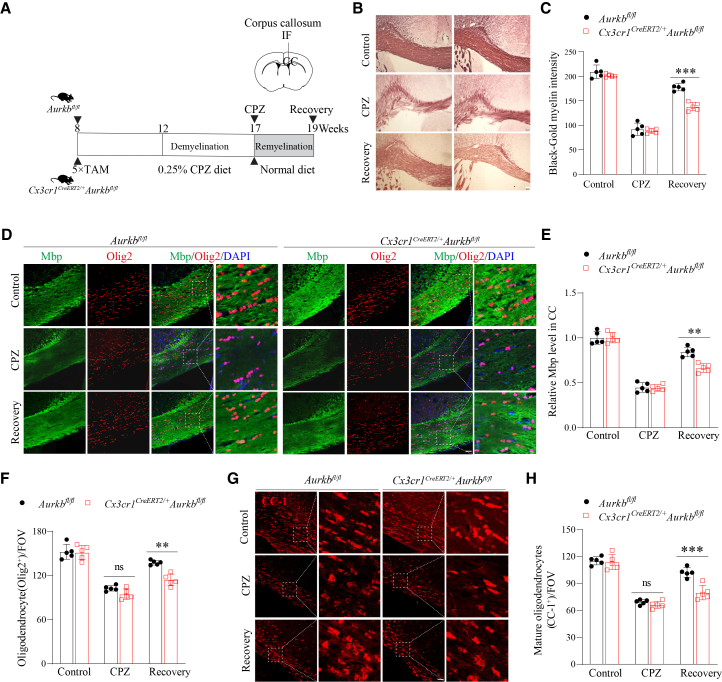
*Aurkb* deficiency in microglia disrupts remyelination and oligodendrocyte density in the CPZ-induced demyelination model (A) Adult *Aurkb*^*fl/fl*^ and *Cx3cr1*^*CreERT2/+*^*Aurkb*^*fl/fl*^ mice (2–3 months old) were *i.p.* injected with TAM for 5 consecutive days. After one month, the mice were fed a 0.25% CPZ-enriched diet for 5 weeks to induce demyelination (CPZ group) or followed by a CPZ-free normal diet for an additional 2 weeks (Recovery group). The corpus callosum (CC) was analyzed by immunofluorescence (IF) and Black-Gold II myelin staining. (B and C) Representative Black-Gold II myelin staining images and quantification of myelin intensity (*n* = 5 mice per genotype per group, Scale bars: 200 μm). (D–F) Representative immunofluorescence analysis and quantification of total Mbp and oligodendrocytes (Olig2^+^), and g-h) representative immunofluorescence analysis and quantification of mature oligodendrocytes (CC-1^+^) (*n* = 5 mice per genotype per group, Scale bars: 50 μm). Data are presented as the mean ± SD. two-way ANOVA with Bonferroni multiple comparisons test in (C, E, F, and H); ∗∗*p* < 0.01 and ∗∗∗*p* < 0.001; ns, not significant compared with the *Aurkb*^*fl/fl*^ group.

To determine the role of Aurkb in microglia during remyelination, we utilized the CPZ-induced demyelination model (Figure 6A). This model employs the copper chelator CPZ to selectively induce oligodendrocyte loss, leading to the robust and reproducible demyelination of the CC, followed by spontaneous remyelination upon CPZ withdrawal.^42^ To circumvent the confounding effects of microglial developmental defects associated with neonatal *Aurkb* deletion, we specifically employed the adult-inducible knockout system for this investigation. Accordingly, adult *Aurkb*^*fl/fl*^ and *Cx3cr1*^*CreERT2/+*^*Aurkb*^*fl/fl*^ mice were *i.p.* injected with TAM for 5 consecutive days. After one month, the mice were fed a 0.25% CPZ-enriched diet for 5 weeks to induce demyelination (CPZ group) or followed by a CPZ-free normal diet for an additional 2 weeks (Recovery group) (Figure 6A).

Black-Gold II staining revealed comparable myelination in the CC of control and *Aurkb-deficient* mice at steady-state conditions (Figures 6B and 6C). After 5 weeks of CPZ treatment, both control and *Aurkb-deficient* mice exhibited demyelination with reduced myelin density (Figures 6B and 6C). However, the myelin density remains significantly reduced in the CC of *Aurkb*-deficient mice during the recovery phase (Figures 6B and 6C). Consistent with this, immunofluorescence analysis demonstrated decreased Mbp, a major component of myelin, and fewer oligodendrocyte lineage cells (Olig2^+^) in *Aurkb*-deficient mice during the recovery phase (Figures 6D–6F). Notably, loss of *Aurkb* in microglia markedly reduced the density of mature oligodendrocytes (CC-1^+^), indicating impaired oligodendrocyte differentiation during remyelination (Figures 6G and 6H). These findings suggest that Aurkb is required for efficient remyelination, likely by supporting oligodendrocyte regeneration and maturation.

### Microglial aurora kinase B deletion impairs phagocytic clearance of myelin debris and zymosan and disrupts autophagic flux

**Figure 7:**
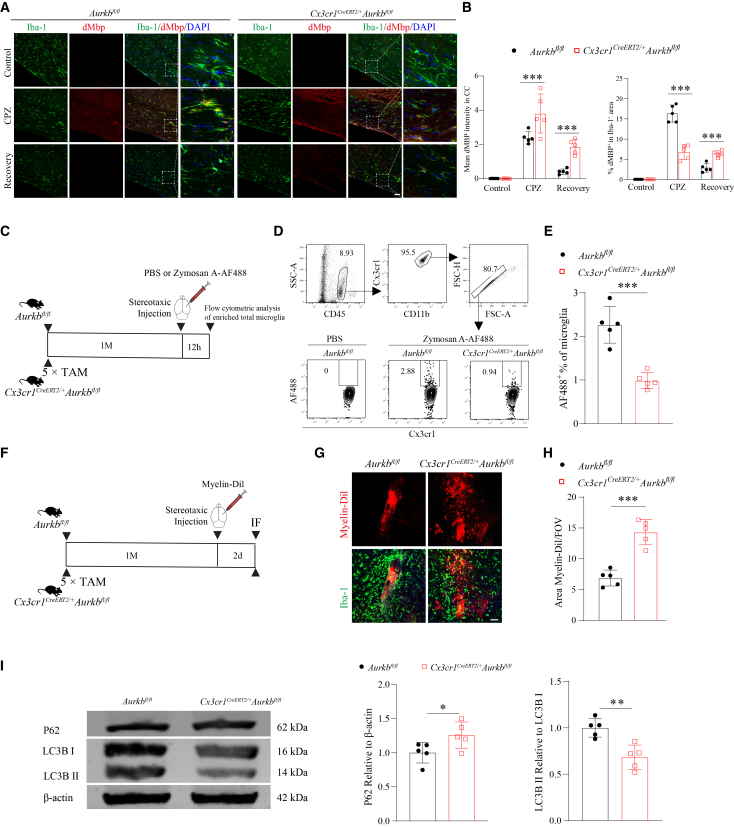
Ablation of *Aurkb* in microglia reduces phagocytic clearance of myelin debris and zymosan and disrupts autophagic flux (A and B) Representative immunofluorescence images and quantification of microglia (Iba-1^+^) and degraded myelin debris (dMbp^+^) in the CC of the control, CPZ, and Recovery groups from *Aurkb*^*fl/fl*^ and *Cx3cr1*^*CreERT2/+*^*Aurkb*^*fl/fl*^ mice (*n* = 5 mice per genotype per group, Scale bars: 50 μm). (C) *In vivo* Zymosan phagocytosis assay: adult *Aurkb*^*fl/fl*^ and *Cx3cr1*^*CreERT2/+*^*Aurkb*^*fl/fl*^ mice were pre-treated with TAM for 5 days. After one month, the mice subsequently received a stereotaxic injection of Zymosan-AF488 or PBS. Microglia from total brains were enriched 12 h post-injection for flow cytometric analysis. (D and E) Representative flow cytometric gating strategy and quantification (E) of microglia phagocytosing Zymosan (AF488^+^) (*n* = 5 mice per genotype). (F) *In vivo* myelin debris phagocytosis assay: Mice received TAM as in (C), followed by the stereotaxic injection of Myelin-Dil. (G and H) Representative immunofluorescence and quantification of (H) phagocytic clearance of Myelin-Dil at 48 h post-injection (*n* = 5 mice per genotype, Scale bars: 50 μm). (I) Western blot (left panel) and quantification (right panel) of P62 and LC3B Ⅰ/Ⅱ in microglia (*n* = 3 mice per genotype). Data are presented as the mean ± SD. two-way ANOVA with Bonferroni multiple comparisons test in (B); Two-tailed unpaired t-tests in (E, H, and I); ∗*p* < 0.05, ∗∗*p* < 0.01, and ∗∗∗*p* < 0.001 compared with the *Aurkb*^*fl/fl*^ group. See Data S1 for uncropped blots.

Given the accumulation of myelin debris in *Aurkb*-deficient mice during the recovery phase, we assessed microglial phagocytosis of myelin debris. Immunofluorescence analysis revealed increased degraded Mbp (dMbp^+^) co-localized with microglia (Iba-1^+^) in the CC of *Aurkb*-deficient mice during both demyelination and remyelination phases (Figures 7A and 7B), suggesting impaired debris clearance. Notably, microglial density in the CC failed to increase during either phase in *Aurkb*-knockout mice, indicating an impaired proliferative or recruitment response to demyelination (Figures 7A and 7B). To further assess microglial phagocytic capacity independent of cell density differences, we performed *in vivo* phagocytosis assays using fluorescently labeled Zymosan (Zymosan-AF488) and myelin debris (myelin-Dil). For this, we used adult *Aurkb*^*fl/fl*^ and *Cx3cr1*^*CreERT2/+*^*Aurkb*^*fl/fl*^ mice and performed analysis one month after tamoxifen induction, when microglial numbers were comparable (Figures 4A and 4B). Flow cytometric analysis revealed a significantly lower percentage of *Aurkb*-deficient microglia internalizing zymosan compared to controls (Figures 7C–7E). Similarly, stereotaxic injection of myelin-Dil revealed markedly reduced phagocytic clearance of myelin-Dil by *Aurkb*-deficient microglia at 48 h post-injection (Figures 7F–7H). Altogether, these data establish that Aurkb is required for maintaining microglial phagocytic function, a key mechanism underlying its role in promoting remyelination.

Given the established role of Aurkb in regulating mTOR signaling in lymphocytes,^23^ and the critical function of the mTOR-autophagy axis in microglial phagocytosis,^24^^,^^25^ we examined whether Aurkb regulates this pathway in microglia. Western blot analysis revealed that *Aurkb* deficiency led to significantly impaired autophagic flux, characterized by increased P62 and decreased LC3B-II/LC3B-I ratio (Figure 7I).

These findings collectively imply that Aurkb may mediate microglial phagocytosis and responses to remyelination via controlling autophagy.

## Discussion

Aurkb is classically known for its role in mitosis, particularly in regulating chromosomal segregation and cytokinesis in proliferating cells.^19^^,^^43^ However, recent evidence suggests that its functions extend beyond cell division, including roles in lymphocyte survival, immune modulation, and tissue repair.^22^^,^^23^^,^^44^^,^^45^^,^^46^^,^^47^ Here, we identify a previously underappreciated role of Aurkb in regulating microglial development, homeostasis, and remyelination in the CNS.

Our data show that Aurkb is essential for microglial expansion during early postnatal development. Microglia-specific deletion of *Aurkb* in neonates led to reduced cell density, impaired proliferation, and increased apoptosis. These findings are consistent with the canonical function of Aurkb in regulating cell mitosis underscore its critical role as a mitotic kinase during microglial expansion. Notably, this requirement extends into adulthood. Despite lower expression in homeostatic microglia, inducible deletion of *Aurkb* resulted in a dystrophic microglial morphology, characterized by reduced branching complexity and a progressive loss of cell density. These results, together with evidence of Aurkb functioning in G_0_/G_1_ quiescent cells,^22^^,^^23^ suggest a dual role for Aurkb in driving mitosis during development and sustaining microglial maintenance in the adult CNS.

Interestingly, *Aurkb*-deficient microglia exhibited transient upregulation of CD68 in the absence of inflammatory stimuli. However, these cells failed to mount a typical response to LPS, showing impaired activation and altered morphology. Considering the established role of Aurkb in regulating mTOR pathway and our finding of impaired autophagy in Aurkb-deficient microglia, we propose that this CD68 elevation likely implies a compensatory expansion of the lysosomal compartment in response to disruptions in autophagic flux, rather than classical immune activation.^48^ Additionally, the shifting profile of microglial density and CD68 expression across the three timepoints mirrors a clear dynamic progression. The data transition from concurrent developmental deficit and acute stress at P13, to a pure stress response at 1-month post-adult *Aurkb* deletion, and finally to chronic homeostatic failure by 3 months, underscoring the essential role of Aurkb in microglial development and homeostasis.

One of the central findings of this study is that Aurkb is critical for microglial clearance of myelin debris in the CPZ-induced demyelination model. *Aurkb*-deficient microglia displayed reduced uptake of myelin and zymosan particles, suggesting defective phagocytosis. Our data further reveal that this is accompanied by impaired autophagic flux, as evidenced by p62 accumulation and a reduced LC3B-II/LC3B-I ratio. We propose that the phagocytic impairment may stem from a combination of cytoskeletal disorganization due to impaired mitosis and defective clearance through the autophagy-lysosomal pathway. On the other hand, both neonatal and adult *Aurkb*-deficient microglia exhibited a dystrophic phenotype, characterized by shortened processes and reduced functional capacity. This morphology resembles that of senescent microglia seen in aging and neurodegenerative diseases.^49^^,^^50^^,^^51^ We speculate that persistent mitotic stress and disrupted homeostasis due to *Aurkb* loss may promote a senescence-like state, which may also contribute to the observed phagocytic dysfunction and delayed remyelination. The resulting accumulation of myelin debris was associated with reduced oligodendrocyte differentiation and impaired remyelination, a pathological feature also observed in chronic MS lesions.^8^^,^^42^^,^^52^^,^^53^ These data indicate that Aurkb-dependent microglial phagocytosis is essential for creating a reparative environment during demyelination.

In summary, this study identifies Aurkb as a key regulator of microglial development, homeostasis, and responses to remyelination. Our findings reveal its dual roles as a mitotic kinase and a modulator of phagocytic and autophagic functions, providing further insights into the mechanisms underlying CNS myelin repair.

### Limitations of the study

While this study establishes a critical role for Aurkb in microglial development, homeostasis, and CNS remyelination, several limitations warrant consideration. First, although we have linked Aurkb deficiency to concomitant defects in phagocytosis and autophagy, our experimental design cannot establish the precise causal relationships between these processes and the ensuing remyelination failure. Future studies employing targeted rescue strategies are needed to elucidate their individual contributions. Second, our conclusions are primarily based on the CPZ-induced demyelination model. Further investigation is required to determine whether Aurkb plays a comparable role in CNS remyelination under more physiological conditions, such as during aging. Studies in aged mouse models would be particularly valuable to address this. Finally, the translational relevance of our findings to human neurodegenerative conditions, including MS and aging-related decline, remains to be fully explored through approaches such as utilizing human microglia-like cells or analyzing postmortem brain tissues.

## Resource availability

### Lead contact

Requests for further information and resources should be directed to and will be fulfilled by the lead contact, Hui Wang (hui.wang@xzhmu.edu.cn).

### Materials availability

*Aurkb*^*fl/fl*^ mice are available from the lead contact upon reasonable request for academic research purposes.

### Data and code availability

• This article analyzes existing, publicly available data, accessible at Gene Expression Omnibus (GEO, GSE121654, GSE123025, GSE207570, GSE204755, and GSE301908).
• All original code has been deposited at GitHub at https://github.com/YelinZhao-A/Aurkb_microglia and is publicly available as of the date of publication.
• Any additional information required to reanalyze the data reported in this article is available from the lead contact upon request.

## Acknowledgments

We sincerely thank Dr. Fuxing Dong from the Public Experimental Research Center for his assistance with the laser scanning confocal microscopy. This work is supported by the 10.13039/100014718National Natural Science Foundation of China (82171791 to H.W.), the 10.13039/100014474Postgraduate Research & Practice Innovation Program of Jiangsu Province (KYCX24_3140 to W.Y. and KYCX25_3227 to D.Z.), the 10.13039/100017360Open Research Project of Key Laboratory of Universities in Jiangsu Province (SZDSYS202202 to S.G.), the 10.13039/100000090Medical Research Project of Jiangsu Provincial Health Commission (ZQ2024032 to. S.G.), the 10.13039/100016105Institutional Research Project of Xuzhou Maternal and Child Health Hospital (XFBYKTY202414 to S.G.), the 10.13039/501100013280Research Project of the Jin Peiying Fund, the 10.13039/100020439Jiangsu Pharmaceutical Association (J2023007 to G.J.), Xuzhou Leading Medical Talent Training Program, and the 10.13039/100007219Jiangsu Provincial Natural Science Foundation Project (BK.20241771 to G.J.).

## Author contributions

Conceptualization: H.W., W.Y., and S.G.; methodology: W.Y. and Y.Z.; software: Y.Z.; formal analysis: W.Y. and Y.Z.; investigation: W.Y., D.X., L.D., D.Z., Q.J., Y.L., S.W., L.L., and H.G.; resources: H.W. S.G., and G.J.; writing: H.W., W.Y., and Y.Z.; supervision: H.W. and S.G.; funding acquisition: H.W., S.G., G.J., W.Y., and D.Z.

## Declaration of interests

The authors declare no competing interests.

## Declaration of generative AI and AI-assisted technologies in the writing process

During the preparation of this work, the authors used DeepSeek to improve grammar and language. After using this tool, the authors reviewed and edited the content as needed and take full responsibility for the content of the publication.

## STAR★Methods

### Key resources table

**Table:** 

REAGENT or RESOURCE	SOURCE	IDENTIFIER
**Antibodies**		

FITC anti-mouse CD45 Antibody, clone 30-F11	BioLegend	Cat# 103108, RRID:AB_312973
PE/Cyanine7 anti-mouse CD45 Antibody, clone 30-F11	BioLegend	Cat# 103114, RRID:AB_312979
APC anti-mouse CX3CR1 Antibody, clone SA011F11	BioLegend	Cat# 149008, RRID:AB_2564492
APC anti-mouse CD11b Antibody, clone M1/70	BioLegend	Cat# 101212, RRID:AB_312795
FITC anti-mouse CD11b Antibody, clone M1/70	BioLegend	Cat# 101206, RRID:AB_312789
PerCP/Cy5.5 anti-mouse CD68 Antibody, clone FA-11	BioLegend	Cat# 137010, RRID:AB_2260046
Rabbit anti-Iba-1 Antibody	Wako	Cat# 019–17409, RRID:AB_839504
Mouse anti-Iba-1 Antibody	Abcam	Cat# ab283319, RRID:AB_2313786
Rat anti-Mouse CD206 Monoclonal antibody, clone MR5D3	Bio-Rad	Cat# MCA2235, RRID:AB_324622
Rabbit anti-Histone H3 Antibody	Proteintech	Cat# 17168-1-AP, RRID:AB_2716755
Mouse anti-phospho-Histone H3(Ser10) Antibody	Cell Signaling Technology	Cat# 9706S, RRID:AB_331748
Mouse anti-Gapdh Antibody	Proteintech	Cat# 60004-1-Ig, RRID:AB_2107436
Mouse anti-β actin Antibody	Proteintech	Cat# 66009-1-Ig, RRID:AB_2687938
Rabbit anti-Aurora B Antibody	Affinity	Cat# AF6475, RRID:AB_2835294
Rat anti-BrdU Antibody, clone BU1/75 (ICR1)Antibody	Abcam	Cat# ab6326, RRID:AB_2313786
Rat anti-CD68 Antibody, clone FA-11 Antibody	Abcam	Cat# ab53444, RRID:AB_869007
Rabbit anti-Olig2 Antibody	Proteintech	Cat# 13999-1-AP, RRID:AB_2157541
Rat Anti-Myelin Basic Protein Monoclonal Antibody, Clone 12	Abcam	Cat# ab7349, RRID:AB_305869
Rabbit anti-CC1 Antibody	Asis Biofarm	Cat# OB-PRB070, RRID: AB_2934254
Rabbit anti-damaged myelin basic protein	Millipore Sigma	Cat# AB5864, RRID: AB_2140351
Rabbit anti-LC3B Antibody	Proteintech	Cat# 81004-1-RR, RRID: AB_2923695
Rabbit anti-P62 Antibody	Proteintech	Cat# 31403-1-AP, RRID: AB_3669966
Donkey anti-Rabbit IgG (H + L) Antibody, Alexa Fluor 488 Conjugated	Thermo Fisher Scientific	Cat# A-21206, RRID:AB_2535792
Donkey Anti-Rabbit IgG (H + L) Polyclonal Antibody, Alexa Fluor 555 Conjugated	Thermo Fisher Scientific	Cat# A-31572, RRID:AB_162543
Goat anti-Rabbit IgG (H + L) Highly Cross-Adsorbed Secondary Antibody, Alexa Fluor™ Plus 647	Thermo Fisher Scientific	Cat# A-32733, RRID:AB_2633282
Goat Anti-Rat IgG (H + L) Antibody, Alexa Fluor 488 Conjugated	Thermo Fisher Scientific	Cat# A-11006, RRID:AB_141373
Goat anti-Mouse IgG (H + L) Cross-Adsorbed Secondary Antibody, Alexa Fluor™ 555	Thermo Fisher Scientific	Cat# A-21422, RRID:AB_2535844
Dylight 800, Goat Anti-Mouse IgG	Abbkine	Cat# A23910, RRID:AB_3661628
Dylight 800, Goat Anti-Rabbit IgG	Abbkine	Cat# A23920, RRID:AB_3661631

**Chemicals, peptides, and recombinant proteins**		

2 × Tap Master PCR Mix	Vazyme	Cat# P112
DL2000 Plus DNA Marker	Vazyme	Cat# MD101
Agaro LE	VICMED	Cat# VIC014G
Mounting Medium with DAPI	Abcam	Cat# ab104139
Brdu	Bestbio	Cat# BB-4261
*In Situ* Cell Death Dection Kit, TMR Red	Roche	Cat# 12156792910
Cuprizone	Sigma-Aldrich	Cat# 14690
Percoll	Cytiva	Cat# GE17-0891-01
eBioscience™ Permeabilization Buffer (10X)	ThermoFisher Scientific	Cat# 00-8333-56
eBioscience™ IC Fixation Buffer	ThermoFisher Scientific	Cat# 00-8222-49
Annexin V	BioLegend	Cat# 640920
Zombie YellowTM Dye	BioLegend	Cat# B296323
RIPA Lysis Buffer	Beyotime Biotechnology	Cat# P0013B
100 × PMSF	Beyotime Biotechnology	Cat# ST506
50 × Protease and phosphatase inhibitor cocktail	Beyotime Biotechnology	Cat# P1045
BCA Protein Assay Kit	Beyotime Biotechnology	Cat# P0012
5 × loading buffer	Beyotime Biotechnology	Cat# P0015L
PAGE Gel Quick Preparation Kit (10%)	Yeasen	Cat# 20325ES62
180 kDa Prestained Protein	Vazyme	Cat# MP102-01-AA
PVDF	Beyotime Biotechnology	Cat# FFP80
Skim milk powder	VICMED	Cat# VIC141
Tween 20	VICMED	Cat# VIC400
Tamoxifen	Solarbio Life Sciences	Cat#10540-29-1
Tissue-Plus O.C.T. Compound	SAKURA Tissue-Tek	Cat# 4583
4% paraformaldehyde	Biosharp	Cat# BL539A
Triton X-100	Meilun Biotech	Cat# 9002-93-1
Bovine Serum Albumin	VICMED	Cat# VIC018
PageRuler Prest Protein Ladder	ThermoFisher Scientific	Cat# 26616
ACK Lysis Buffer	N/A	Homemade
Orthoboric acid, sodium salt	Sangon Biotech	Cat# A500832
Zymosan A (S. cerevisiae) BioParticles™, Alexa Fluor™ 488 conjugate	ThermoFisher Scientific	Cat# Z23373
LPS	Sigma-Aldrich	Cat# L6143
Dil	Yuanye	Cat# S25170
Black-Gold Ⅱ Myelin	Biosensis	Cat# BSS -TR-100-BG
PBS(10x)	KeyGEN BioTECH	Cat# KGB50011

**Deposited data**		

scRNA-seq data of microglia from various developmental stages (E14.5-P570)	N/A	https://www.ncbi.nlm.nih.gov/geo/query/acc.cgi?acc=GSE121654
scRNA-seq data of microglia from various developmental stages (E14.5-P60)	N/A	https://www.ncbi.nlm.nih.gov/geo/query/acc.cgi?acc=GSE123025
scRNA-seq data of microglia from the CPZ-induced demyelination	N/A	https://www.ncbi.nlm.nih.gov/geo/query/acc.cgi?acc=GSE207570
scRNA-seq data of microglia from the CPZ-induced demyelination and remyelination	N/A	https://www.ncbi.nlm.nih.gov/geo/query/acc.cgi?acc=GSE204755
scRNA-seq data of frozen autopsy brain tissues of control and MS subjects	N/A	https://www.ncbi.nlm.nih.gov/geo/query/acc.cgi?acc=GSE301908

**Experimental models: Organisms/strains**		

Mouse:*Aurkb*^*fl/fl*^(C57BL/6J background)	Cyagen Biosciences Inc	N/A
Mouse:*Cx3cr1*^*Cre*^	The Jackson Laboratory	JAX: 025524
Mouse:*Cx3cr1*^*CreERT2*^	The Jackson Laboratory	JAX: 021160

**Software and algorithms**		

FlowJo software	BD Biosciences	https://www.flowjo.com/
ImageJ software	N/A	https://imagej.net/Welcome
illustrator	Adobe	https://www.adobe.com/
Prism	GraphPad	https://www.graphpad.com/
R (version 4.2.2)	N/A	http://www.r-project.org/

**Other**		

FACSAria III, flow cytometry & cell sorter	BD Biosciences	https://www.bdbiosciences.com/en-se/products/instruments/flow-cytometers/research-cell-sorters/bd-facsaria-iii
Ultrasonic Homogenizer	Scientz Z	https://www.scientzbio.com/
STELLARIS 5 Confocal scanning microscopy	Leica	https://www.leica-microsystems.com/
NanoDrop 2000 Spectrophotometer	ThermoFisher Scientific	https://www.thermofisher.com/order/catalog/product/ND-2000
Frozen microtome	Leica	Cat# CM1950
Syringe pump	Quintessential stereo-taxic injector, Stoelting	Cat# 53311
Mouse stereotaxic Frame	RWD	Cat# 51730U
Stereotaxic apparatus	RWD	Cat# 68507
Bio-Spin P-6 gel Columns, Tris Buffer	Bio-Rad	Cat# 7326227
LSM710/STELLARIS 5 Confocal scanning microscopy	Zeiss/Leica	https://www.leica-microsystems.com/

### Experimental model and study participant details

#### Animals

*Cx3cr1*^*Cre*^ mice (Stock ID: 025524) and *Cx3cr1*^*CreERT2*^ mice (Stock ID: 021160) on the C57BL/6 genetic background were obtained from the Jackson Laboratory. *Aurkb*^*fl/fl*^ mice on the C57BL/6J genetic background were generated by Cyagen Biosciences Inc. using CRISPR- Cas9 technology. To construct these mice, a targeting vector containing homologous arms and *loxP* sites flanking exons 2–6 of *Aurkb* was generated by PCR using the BAC clone RP24-281K2 as a template. Mouse production was performed by co-injecting ribonucleoprotein complexes and the targeting vector into fertilized zygotes.

*Aurkb*^*fl/fl*^ mice were crossed with *Cx3cr1*^*Cre*^ or *Cx3cr1*^*CreERT2*^ to generate control mice *Aurkb*^*fl/fl*^, and microglia-specific *Aurkb* knockout mice, *Cx3cr1*^*Cre/+*^*Aurkb*^*fl/fl*^ and *Cx3cr1*^*CreERT2/+*^*Aurkb*^*fl/fl*^. For inducible *Aurkb* knockout, *Cx3cr1*^*CreERT2/+*^*Aurkb*^*fl/fl*^ mice and control mice *Aurkb*^*fl/fl*^ were injected with tamoxifen (TAM, 75 mg/kg) for three consecutive days at postnatal day 1 and for five consecutive days at 2–3 months of age.

All experimental mice were maintained and bred under specific pathogen free (SPF) conditions at the Laboratory Animal Center of Xuzhou Medical University. Animal experiments were conducted in full compliance with the Institutional Animal Care and Use Guidelines (IACUC) and approved after formal review by the Animal Experimental Ethics Committee of Xuzhou Medical University (Approval No. 202211S011). Mice were housed in plastic cages under controlled environmental and lighting conditions (12-h light/dark cycle), with *ad libitum* access to food and water. All experiments were performed on age- and sex-matched littermates with specific ages detailed in figure legends. Data from male and female mice were pooled for analysis since no sex-based differences were observed.

### Method details

#### LPS-induced inflammation model

To establish LPS-induced inflammation model^54^*,* 8-week-old *Aurkb*^*fl/fl*^ and *Cx3cr1*^*CreERT2/+*^*Aurkb*^*fl/fl*^ mice were injected with PBS or LPS from Salmonella enterica (1 mg/kg, Sigma-Aldrich, Cat: L6143). Twenty-four hours post injection, mice were euthanized for the analysis of microglial activation using immunofluorescence analysis of the colocalization of CD68 with Iba-1.

#### Cuprizone-induced demyelination model

To establish a cuprizone (CPZ)-induced demyelination model, adult *Aurkb*^*fl/fl*^ and *Cx3cr1*^*CreERT2/+*^*Aurkb*^*fl/fl*^ littermates (2–3 months old) were *i.p.* injected with TAM (75 mg/kg) for 5 consecutive days. One month post TAM injection, these mice were fed a standard diet containing 0.25% cuprizone (Sigma-Aldrich, Cat: 14690) for 5 weeks (CPZ group) to induce demyelination.^55^ To assess remyelination, a subset of CPZ-treated mice was returned to a normal diet for an additional two weeks (recovery group). The corpus callosum (CC) of the mice subjected to CPZ-induced demyelination and remyelination were collected for Black gold II myelin staining and immunofluorescence analysis.

#### *In vivo* BrdU assay analysis

*Aurkb*^*fl/fl*^ and *Cx3cr1*^*CreERT2/+*^*Aurkb*^*fl/fl*^ littermates at postnatal day 10 (P10) received intraperitoneal (*i.p.*) injections of 5-bromo-2′-deoxyuridine (BrdU, 100 μL, 10 mg/mL) administered in three consecutive bolus doses at 24-h intervals.^56^ Twenty-four hours after the final injection, the animals were euthanized for microglial proliferation analysis using immunofluorescence staining.

#### Immunofluorescence analysis

Immunofluorescence analysis was conducted as described previously.^57^ Mice were anesthetized with pentobarbital sodium and subjected to transcardial perfusion with 4% paraformaldehyde (PFA). The brains were carefully dissected and post-fixed in 4% PFA overnight. Subsequently, the tissue underwent graded sucrose cryoprotection, first in 15% sucrose for 24 h and then in 30% sucrose for an additional 24 h. Coronal brain sections (20 μm thick) were obtained using a freezing microtome (Leica CM1950). The sections were incubated in blocking buffer containing 10% goat serum, 1% bovine serum albumin (BSA), and 0.3% Triton X- in phosphate-buffered saline (PBS) for 2 h at room temperature. Next, the sections were incubated overnight at 4°C with a mixture of primary antibodies diluted in blocking buffer. The following primary antibodies were used: rabbit anti-Iba-1 antibody (1:500, Wako, Cat: 019–19741), mouse anti-Iba-1 antibody (1:400, Abcam, Cat: ab283319), rat anti-CD206 antibody (1:200, Bio-Rad, Cat: MCA2235), rat anti-BrdU antibody (1:400, Abcam, Cat: ab6326), mouse anti-phospho-Histone H3 (Ser10) antibody (1:200, Cell Signaling Technology, Cat: 9706S), mouse anti-APC (1:100, CC-1, Merck, Cat: OP80), rat anti-myelin basic protein (Mbp) monoclonal antibody (1:500, Abcam, Cat: ab7349), rabbit anti-degraded myelin basic protein (dMbp) antibody (1:2000, Millipore Sigma, Cat: AB5864), rabbit anti-Olig2 antibody (1:500, Proteintech, Cat: 13999-1-AP) and rat anti-CD68 antibody (1:500, Abcam, Cat: ab53444). After washing five times with PBS at room temperature, the sections were incubated with species-specific secondary antibodies conjugated to Alexa Fluor dyes: Alexa Fluor 488 (1:500, ThermoFisher Scientific, Cat: A-21206/A-11006), Alexa Fluor 555 (1:500, ThermoFisher Scientific, Cat: A-31572/A-21422) and Alexa Fluor 647 (1:500, ThermoFisher Scientific, Cat: A-32733), for 2 h at room temperature. TUNEL staining was performed according to the manufacturer’s protocol (Roche, Cat: 12156792910) following the removal of secondary antibodies. After washing the sections five times with PBS, they were mounted with DAPI mounting medium (Abcam, Cat: ab104139) and confocal laser scanning microscope (CLSM, Leica STELLARIS 5, Germany).

#### Microglial morphological analysis

The experimental procedures strictly followed established methodologies from prior literature.^58^^,^^59^^,^^60^ Briefly, micrographs underwent preprocessing through sequential steps: raw images were first converted to 8-bit format, followed by brightness/contrast optimization to enhance visualization of microglial branching architecture. Pixel noise was subsequently eliminated using the despeckling algorithm. After optimal threshold adjustment, we sequentially executed despeckling and outlier removal functions to refine binary images, which were then skeletonized for morphological quantification. Total cell counts per micrograph were manually quantified. Comprehensive morphometric analyses included measurements of total branch length per image, enumeration of branch endpoints, and calculation of total branch intersections. For identical isolated cells subjected to both fractal and skeleton analyses. Sholl analysis was performed within ImageJ by establishing concentric circle radii extending from the soma centroid to the terminal point of the longest dendritic branch, thereby defining analytical boundaries. Analytical methodology employed quantitative assessment using biological replicates: three microglial cell samples per mouse were analyzed on average, followed by comparative analysis across animals.

#### Flow cytometric analysis

Prior to flow cytometric analysis, single-cell suspensions were prepared from mouse brains.^61^ Brains from newborn and adult mice were minced and subjected to gentle mechanical homogenization. The resulting suspension was layered onto a 40% Percoll gradient and centrifuged at 800 g for 30 min at room temperature to enrich microglia.

For the analysis of CD68 expression, microglia-enriched cell suspensions were labeled with antibodies specific for microglial surface markers (CD45^lo^Cx3cr1^+^CD11b^+^) for 15 min at room temperature. Subsequently, cells were fixed and permeabilized, followed by incubation with anti-mouse CD68 antibody (clone FA-11, BioLegend, Cat: 137010) for 30 min at room temperature. After staining, cells were washed with 1× permeabilization buffer to remove unbound antibodies prior to flow cytometric analysis.

To analyze microglial apoptosis, microglia-enriched cell suspensions were labeled with antibodies against microglia markers (CD45^lo^Cx3cr1^+^CD11b^+^) and APC-Annexin V (BioLegend, Cat: 640920) diluted in 1 × Annexin-V Binding Buffer for 30 min, followed by washing with 1 × Annexin-V Binding Buffer prior to flow cytometric analysis.

#### Western blot

For protein analysis, minced mouse brain tissues were subjected to gentle mechanical homogenization.^62^ The resulting suspension was layered onto a 40% Percoll gradient and centrifuged at 800 × g for 30 min at room temperature to enrich microglia. The microglia-enriched cell suspensions were lysed using RIPA lysis buffer (Beyotime Biotechnology, Cat: P0013B) supplemented with a protease and phosphatase inhibitor cocktail (Beyotime Biotechnology, Cat: P1045). The lysate was centrifuged at 12000 g for 15 min, and the supernatant was collected. Protein concentration was quantified using a BCA protein assay kit (Beyotime Biotechnology, Cat: P0012S). Equal amounts of protein from each sample were mixed with 5× loading buffer, heated at 100°C for 5 min, and loaded onto an SDS-PAGE gel. After electrophoresis, proteins were transferred onto a nitrocellulose membrane. The membrane was subsequently blocked with 5% bovine serum albumin (BSA) at room temperature for 2 h to prevent nonspecific binding and stained with rabbit anti-Aurora B antibody (1:1000, Affinity, Cat: AF6475), rabbit anti-H3 antibody (1:5000, Proteintech, Cat: 17168-1-AP), mouse anti-phospho-Histone H3 (Ser10) (1:1000, Cell Signaling Technology, Cat: 9706S), rabbit anti-LC3B antibody (1:1000, Proteintech, Cat: 81004-1-RR), rabbit anti-P62 antibody (1:1000, Proteintech, Cat: 31403-1-AP), mouse anti-Gapdh antibody (1:5000, Proteintech, Cat: 60004-1-Ig) and mouse anti-β-actin antibody (1:5000, Proteintech, Cat: 66009-1-Ig) at 4°C for overnight. The nitrocellulose membrane was washed three times with Tris-buffered saline with Tween 20 (TBST) for 5 min each. Subsequently, the membrane was incubated with Goat Anti-Mouse/Rabbit IgG Dylight 800 secondary antibody (1:10,000 dilution; Abbkin, Cat: A23910/A23920) at room temperature for 2 h. After the final washes, the membrane was scanned using a Western blot imaging system (Odyssey CLXL1-CDR) for signal detection. The results are expressed as the ratio of target protein to internal control (target protein/internal control), with the mean value of this ratio in the indicated control group designated as 1 unit for quantitative normalization.

#### *In vivo* phagocytosis assays

CNS myelin debris was prepared as previously described and conjugated to 1,1′-dioctadecyl-3,3,3′,3′-tetramethylindocarbocyanine perchlorate (Dil).^63^^,^^64^ To assess microglial phagocytic activity *in vivo*, *Aurkb*^*fl/fl*^ and *Cx3cr1*^*CreERT2/+*^*Aurkb*^*fl/fl*^ *mice* (10–12 weeks old) were *i.p.* injected with TAM (75 mg/kg) for 5 consecutive days to induce conditional *Aurkb* knockout. Under deep anesthesia, 1 μL of Dil-conjugated myelin debris (Myelin-Dil, 20 mg/mL) or Zymosan A conjugated with Alexa Fluor 488 (Zymosan-AF488, 2 mg/mL, ThermoFisher Scientific, Cat: Z23373) was stereotaxically injected into the target brain region using an RWD Life Science stereotactic apparatus (Model 68507). Coordinates for injection were determined based on the Paxinos mouse brain atlas (AP: −1.5 mm, ML: ±1.0 mm, DV: −2.2 mm from bregma).

For immunofluorescence analysis of the clearance of phagocytic target material, 48 h post-injection, mice were transcardially perfused with ice-cold PBS followed by 4% PFA for tissue fixation. The entire injection site was dissected and coronally sectioned into 40 μm-thick slices using a Leica VT100S vibratome. For immunostaining, thick slices were incubated with rabbit anti-Iba-1 antibody (1:500, Wako, Cat: 019–19741), followed by incubation with Alexa Fluor 488-conjugated secondary antibodies. Nuclei were counterstained with DAPI.

To analyze microglial uptake of Zymosan-AF488, mice were euthanized and transcardially perfused with PBS 12 h post-injection. Brain tissues were harvested and processed into single-cell suspensions. Microglia were enriched using a 40% Percoll density gradient and analyzed by flow cytometry. Phagocytosing microglia were identified based on gating for CD45^lo^Cx3cr1^+^CD11b^+^ and fluorescence from internalized Zymosan-AF488.

#### Black-Gold II myelin staining

To specifically visualize intact myelin in CNS tissues, the Black-Gold II (BGII) Myelin staining system (Biosensis, Cat. BSS-TR-100-BG) was used.^65^ BGII is a lipophilic aurophosphate complex that selectively binds to myelin phospholipids, enabling high-contrast detection of myelinated fibers. Using a Leica CM1950 cryostat, 20-μm-thick coronal brain sections were prepared on gelatin-coated slides. The slides were oven-dried at 60°C for 30 min to ensure complete dehydration, followed by rehydration in deionized water for 2 min. Sections were then immersed in a staining solution at 60°C for 12 min. After three sequential 5-min washes in deionized water, slides were transferred to a stop solution for 2 min. Subsequently, slides were dehydrated through a graded ethanol series (70%, 95% and 100%, 3 min each), cleared in xylene for 3 min, and coverslipped using a neutral balsam mounting medium. Brightfield microscopy was used for imaging and analysis of myelinated structures.

#### Single-cell RNA sequencing data analysis

Public single-cell RNA sequencing (scRNA-seq) datasets from mouse microglia at different developmental stages (GSE121654, GSE123025), the cuprizone (CPZ)-induced demyelination model (GSE207570, GSE204755) and frozen autopsy brain tissues of control and MS subjects (GSE301908) were retrieved from the Gene Expression Omnibus (GEO).^27^^,^^28^^,^^29^^,^^66^ The raw count matrices were analyzed using Seurat v4.0.4 to investigate *Aurkb* and *Mki67* expressions. Briefly, quality control was performed to exclude low-quality cells with over 20% mitochondrial gene content or fewer than 200 detected genes. All samples were merged following the standard Seurat integration workflow. Each sample was individually normalized, and the top 2,000 highly variable genes were selected for integration and subsequent analysis. Dimensionality reduction was performed using principal component analysis (PCA), with the top 15 principal components retained, followed by t-distributed stochastic neighbor embedding (t-SNE) for visualization. *Aurkb* and Mki67 expression in microglia, as defined by the original study’s cell type annotations, was visualized using the *FeaturePlot* function, violin or scatterplots.

### Quantification and statistical analysis

Statistical analyses were conducted using GraphPad Prism software (version 10.1.2) and R (version 4.2.2). For comparisons among multiple groups, two-way analysis of variance (ANOVA) with a Bonferroni’s post hoc test was employed, while comparisons between two groups were performed using the two-way Student’s *t* test. Sholl analysis data, comprising repeated measurements, were analyzed using linear mixed-effects models for continuous data and negative binomial generalized linear mixed-effects models for count data with the *lme4* package (version 1.1–33) in R (version 4.2.2) to account for within-animal correlation. *p* values were adjusted via the false discovery rate (FDR) method. All statistical details including the exact value of sample size (n, representing independent biological replicates) and the statistical tests used are reported in the corresponding figure legends. Data are presented as the mean ± SD. Differences were considered significant for *p* < 0.05 (∗*p* < 0.05, ∗∗*p* < 0.01, ∗∗∗*p* < 0.001).
