# Supplementary material for: Aurkb deficiency disrupts microglial development, homeostasis and hinders remyelination following cuprizone-induced demyelination
Source: iScience. 2026 Jan 20;29(2):114718. doi: 10.1016/j.isci.2026.114718 (PMC12907124; doi:10.1016/j.isci.2026.114718)

iScience, Volume *29*

Supplemental information

Aurkb deficiency disrupts microglial development, homeostasis and hinders remyelination following cuprizone-induced demyelination

Weixing Yan, Dong Xiang, Li Du, Di Zhu, Qi Jia, Yuting Liu, Siyu Wang, Li Liu, Haihao Guan, Yelin Zhao, Guan Jiang, Sijia Gao, and Hui Wang

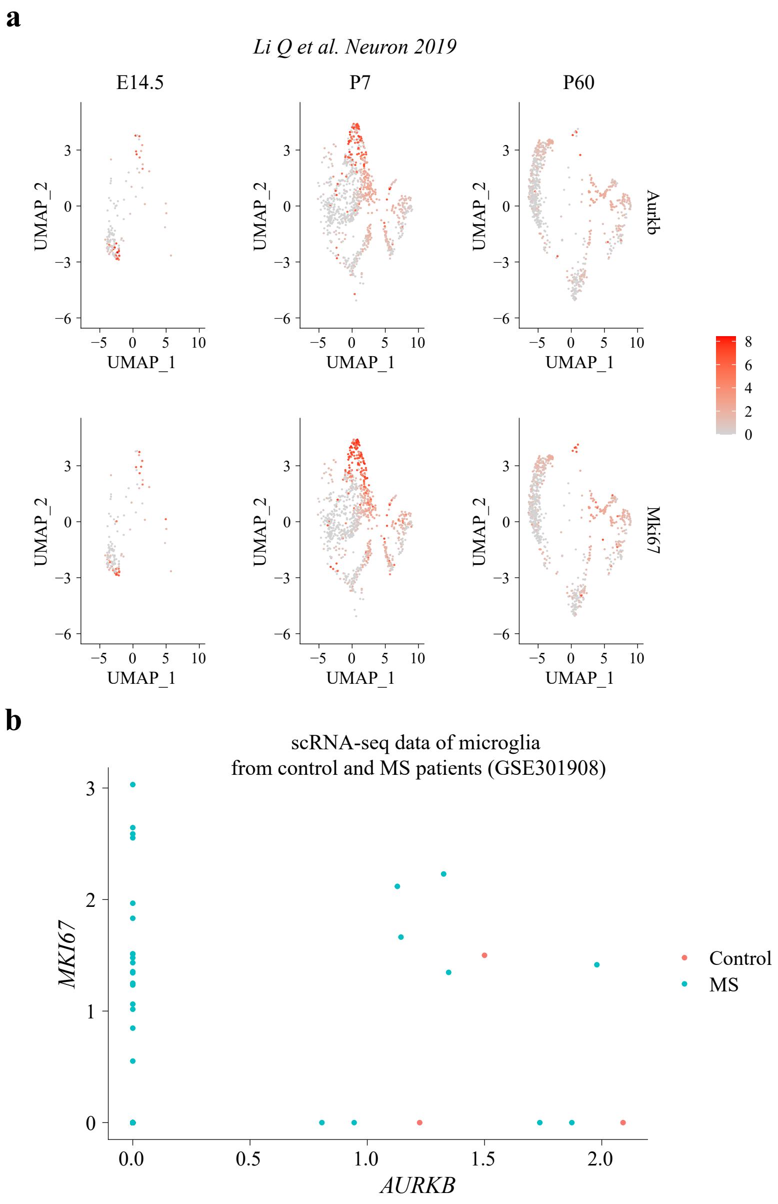

Figure S1. Co-expression of *AURKB* and *MKI67* in microglia during development and in multiple sclerosis.

a) UMAP visualization of *Aurkb* and *Mki67* expression in mouse microglia at E14.5, P7 and P60 (scRNA-seq dataset GSE123025).

b) Scatter plot showing the co-expression of increased *AURKB* and *MKI67* in human microglia from control and multiple sclerosis (MS) cohorts (scRNA-seq dataset GSE301908).

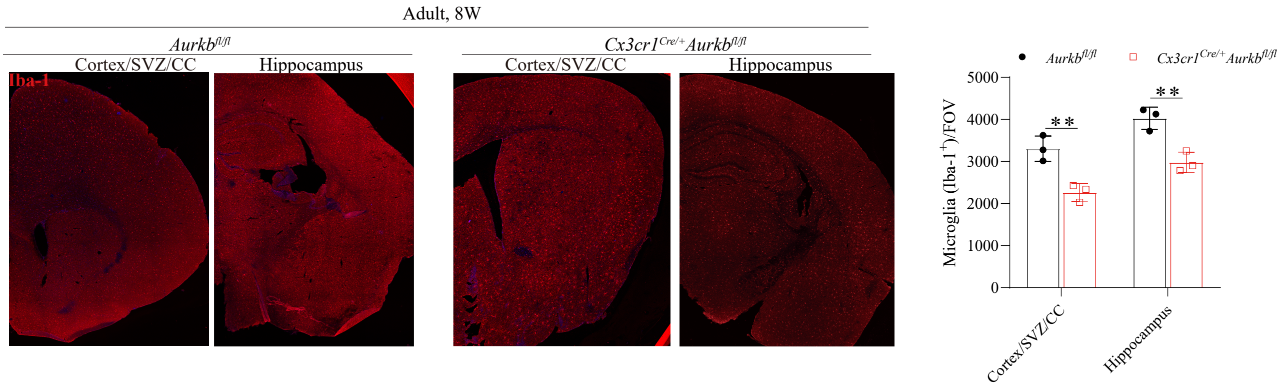

Figure S2. ***Aurkb*** **deficiency reduces microglial density and induces dystrophy in adult mice.**
Representative images of the hemibrain with quantification of Iba-1^{+} microglial density in the SVZ and hippocampal regions of adult *Aurkb*$^{fl/fl}$ and *Cx3cr1*$^{Cre/+}$*Aurkb*$^{fl/fl}$ littermates (n = 3 mice per genotype). Data were presented as the mean ± SD.Two-way ANOVA with Bonferroni multiple comparisons test. **$P < 0.01$ compared with the *Aurkb*$^{fl/fl}$ group.

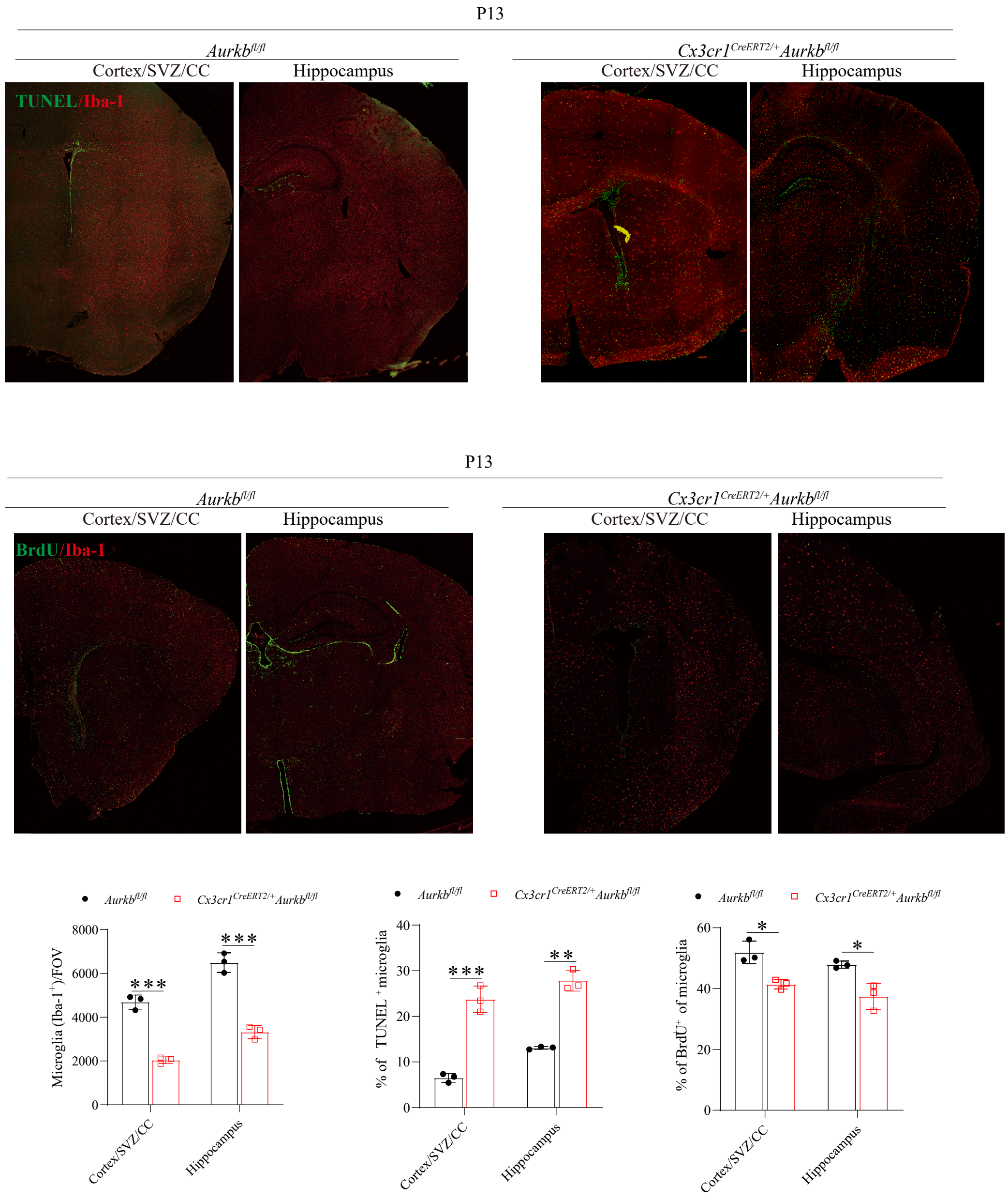

Figure S3. Neonatal *Aurkb* deletion disrupts microglial development.
Neonatal *Aurkb*$^{fl/fl}$ and *Cx3cr1*$^{CreERT2/+}$*Aurkb*$^{fl/fl}$ littermates (n = 3 mice per genotype) were *i.p.* injected with TAM for 3 consecutive days at P1-P3 and sacrificed at P13. Representative hemibrain images with quantification of proliferating microglia (Iba-1^{+}BrdU^{+}) and apoptotic microglia (Iba-1^{+}TUNEL^{+}) in the SVZ andhippocampus. Data were presented as the mean ± SD. Two-way ANOVA with Bonferroni multiple comparisons test. *$P < 0.05$, **$P < 0.01$, ***$P < 0.001$ compared with the *Aurkb*$^{fl/fl}$ group.

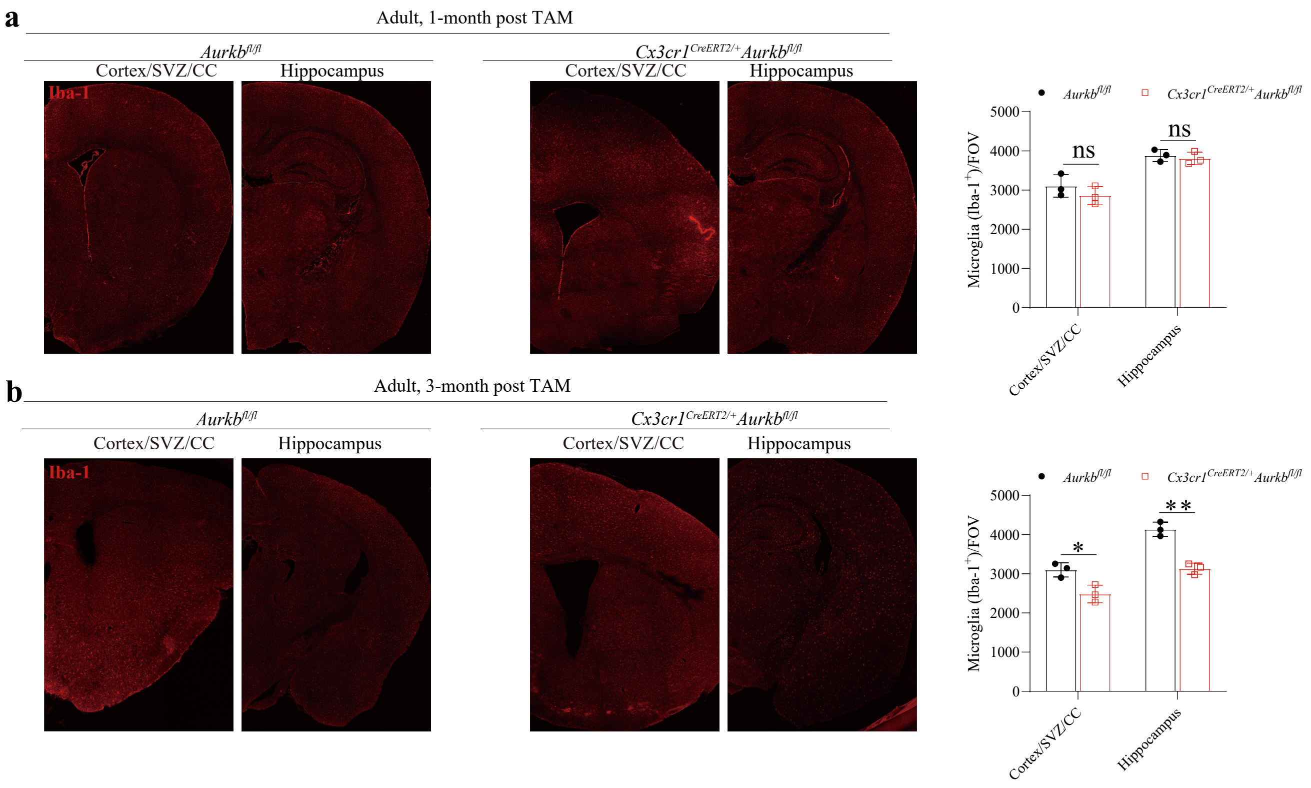

Figure S4. *Aurkb* ablation in adulthood impairs microglial homeostasis.
Adult *Aurkb*$^{fl/fl}$ and *Cx3cr1*$^{CreERT2/+}$*Aurkb*$^{fl/fl}$ littermates (n = 3 mice per genotype) were *i.p.* injected with TAM for 5 consecutive days, followed by tissue collection at 1-month and 3-month post TAM induction. Representative hemibrain images with quantification of Iba-1^{+} microglial density in the SVZ and hippocampal regions at **a**) 1-month and **b**) 3-month post-tamoxifen induction. Data are presented as the mean ± SD. Two-way ANOVA with Bonferroni multiple comparisons test in (**a**, **b**). *$P < 0.05$, **$P < 0.01$; ns, not significant compared with the *Aurkb*$^{fl/fl}$ group.

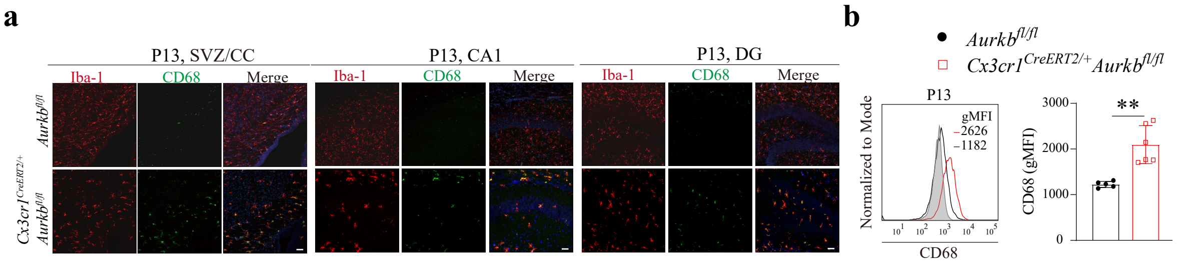

Figure S5. Neonatal ablation of *Aurkb* transiently elevates CD68 in infant microglia.
Neonatal *Aurkb*$^{fl/fl}$ and *Cx3cr1*$^{CreERT2/+}$*Aurkb*$^{fl/fl}$ littermates (n = 5 mice per genotype) were *i.p.* injected with TAM for 3 consecutive days at P1-P3, followed by tissue collection at P13. **a**) Representative immunofluorescence analysis of CD68 in microglia (Iba-1^{+}). Scale bar: 50μm. **b**) Flow cytometric analysis of intracellular CD68 in microglia. Gray histogram: isotype control. gMFI, geometric mean fluorescence intensity. Data were presented as the mean ± SD. Two-tailed unpaired t-tests in (**b**).**$P < 0.01$ compared with the *Aurkb*$^{fl/fl}$ group.

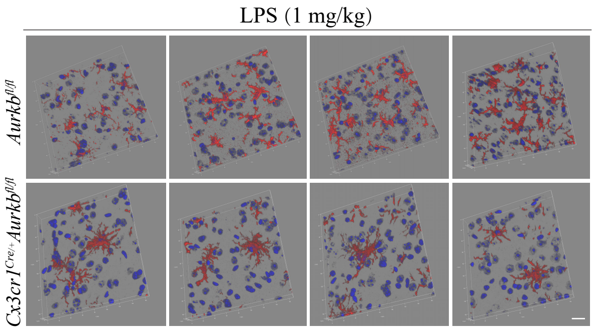

Figure S6 Microglial morphology in an LPS-induced inflammation model. Representative confocal images of microglia from LPS-challenged *Aurkb*$^{fl/fl}$ and *Cx3cr1*$^{Cre/+}$*Aurkb*$^{fl/fl}$ littermates. Images are representative of 5 independent littermate pairs. Scale bar: 20 μm.

Data S1

Figure 1

Marker :
Vicmed(VPM003)

b

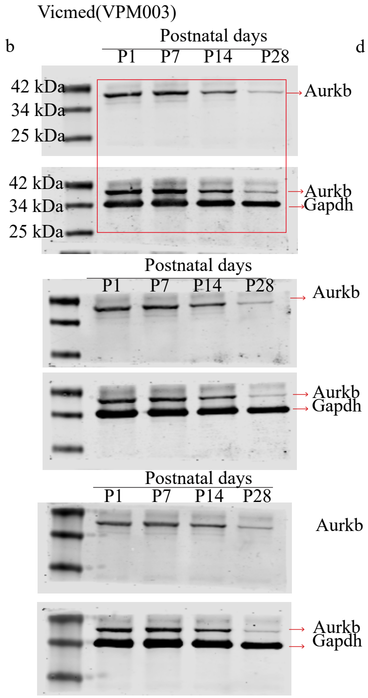

Marker :
Vazyme(MP102-01)

d

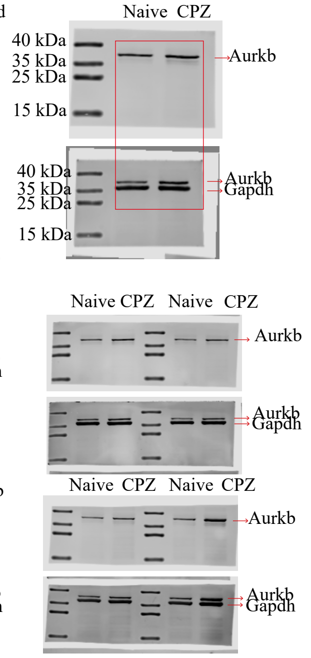

Figure 2

Marker :
Vazyme(MP102-01)

b

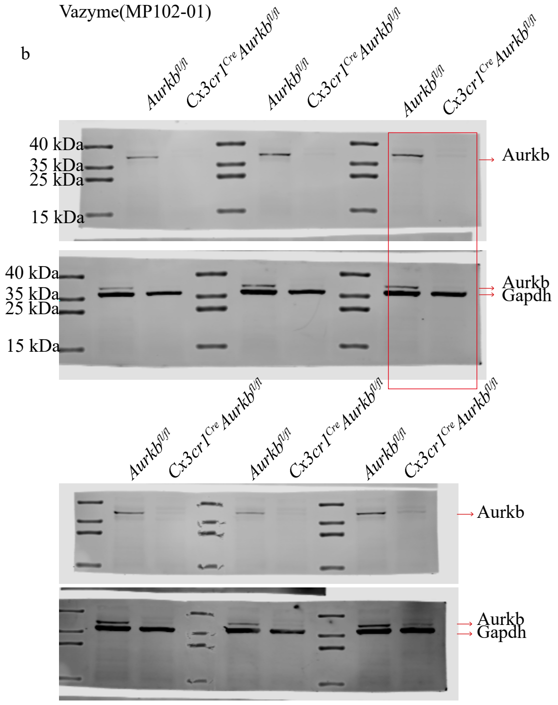

Figure 3

c

Marker : Vicmed(VPM003)

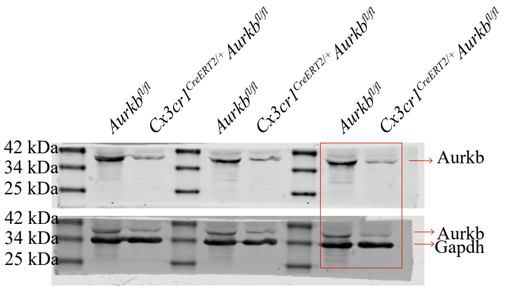

g

Marker :
Vazyme(MP102-01)

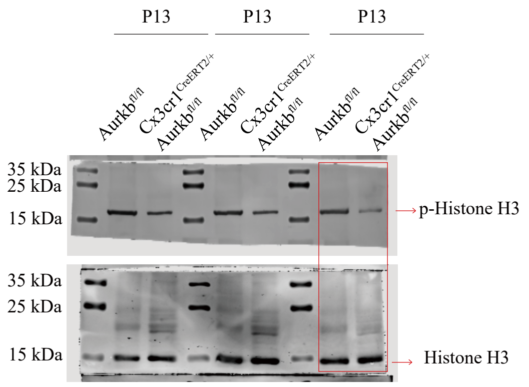

Figure 7

i

Marker :
Vazyme(MP102-01)

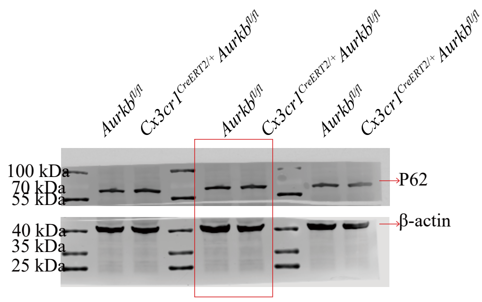

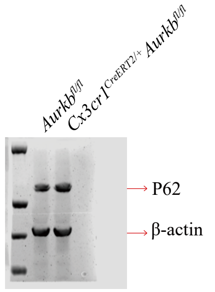

g

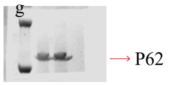

Marker : Vazyme
(MP102-01)

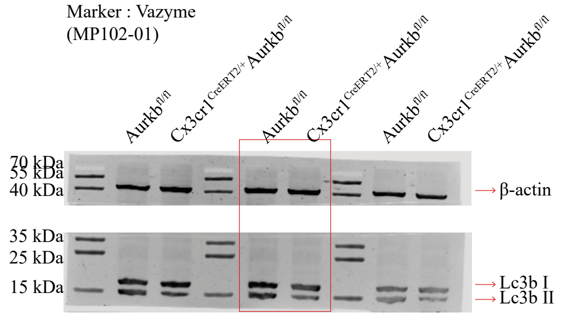

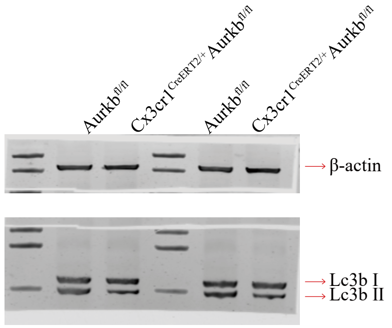

Supplement: Document S1. Figures S1–S6 and Data S1 [file mmc1.pdf]
